# Pharmacokinetics of micafungin in patients with severe burn injuries

**DOI:** 10.1186/cc10667

**Published:** 2012-03-20

**Authors:** J Sasaki, S Kishino, N Aikawa, S Hori

**Affiliations:** 1 Keio University School of Medicine, Tokyo, Japan; 2 Meiji Pharmaceutical University, Tokyo, Japan

## Introduction

Micafungin (MCFG), an echinocandin antifungal agent, exhibits more potent antifungal activity against a broad spectrum of clinically important Candida and Aspergillus species [1]. Few studies have reported the pharmacokinetics (PK) of antifungal agents in patients with burn injuries. A purpose of this study is to characterize the PK of MCFG in severe burn patients.

## Methods

Eight severe burn patients within 14 days after injuries (M:F = 5:3, 19 to 85 years old, 35 to 85% total body surface area) were treated with MCFG (200 to 300 mg, 3.45 to 4.49 mg/kg) once daily by intravenous infusion over 1 hour. The MCFG concentrations in the plasma at the end of the initial administration of MCFG (P1), just before the second dosing (T1), at the end of the fourth dosing (P4), and just before the fifth dosing (T4) were determined, and were compared with the reported values in health volunteers [2]. MCFG concentrations in the burn eschar at T1 and T4 were also measured.

## Results

The plasma concentrations of MCFG per dose normalized with body weight (C/D) at P1, T1, P4, and T4 were 1.37 to 6.28, 0.51 to 1.38, 3.20 to 6.46, and 0.65 to 2.18 (μg/ml)/(mg/kg), respectively, indicating marked interindividual differences. These values were comparable with or slightly lower than the reported values in healthy volunteers (P1: 5.7, T1: 1.3, T4: 2.1). The MCFG concentrations in the burn eschar of three patients at T1 and T4 were <0.1 to 3.98 and 1.10 to 14.81 μg/ml, respectively. Most of MCFG concentrations in the plasma and burn eschar were higher than the reported MIC_90 _of MCFG against clinically important Candida and Aspergillus species. There was no correlation between the laboratory parameters of liver/kidney function and the plasma C/D of MCFG.

## Conclusion

The plasma concentrations of MCFG in patients with severe burn injuries were comparable with or slightly lower than the reported values in healthy volunteers. In addition, MCFG seems to be capable of penetrating burn eschar.
